# Molecular Modeling and Preliminary Clinical Data Suggesting Antiviral Activity for Chlorpheniramine (Chlorphenamine) Against COVID-19

**DOI:** 10.7759/cureus.20980

**Published:** 2022-01-06

**Authors:** Shaun D Black

**Affiliations:** 1 Chemistry and Biochemistry, University of Texas at Tyler, Tyler, USA

**Keywords:** sars-cov-2, covid-19, clinical findings, molecular modeling, antiviral agents, chlorpheniramine maleate

## Abstract

Chlorpheniramine maleate, a widely used over-the-counter antihistamine, has been identified as a structural analog of aminoquinolines known to possess antiviral activity against the Betacoronavirus severe acute respiratory syndrome coronavirus-2 (SARS-CoV-2) that causes coronavirus disease 2019 (COVID-19). Structural similarities include the chlorophenyl group, pyridine ring, alkyl sidechain, and terminal tertiary amine; the comparison of aqueous energy-minimized structures indicates significant three-dimensional similarity as well. Preliminary clinical evidence supports these conclusions. The present study suggests that chlorpheniramine possesses antiviral activity against COVID-19.

## Introduction

The coronavirus pandemic of 2019-2022 has caused over 5,300,000 deaths in over 272,000,000 confirmed cases by late 2021; the United States of America has been the most direly affected of all countries in the world, suffering nearly 15% of all fatalities [1]. The etiologic agent is the Betacoronavirus severe acute respiratory syndrome coronavirus-2 (SARS-CoV-2) that causes coronavirus disease 2019 (COVID-19), first identified in late 2019 in Wuhan, China [2]. Because the world population is naïve to this novel virus, extraordinary mortality has been experienced, and the need for effective therapeutics remains urgent.

Two related Betacoronaviruses have caused widespread mortality and morbidity in the recent past. The SARS-CoV-1 (“SARS”) outbreak of 2002-2004 began in the Guangdong province of southern China [2-5] and has been traced through Asian palm civets to cave-dwelling horseshoe bats [6]. The SARS virus caused a “severe acute respiratory syndrome” in 8,422 victims with a case mortality rate of 9.7% [5]. The Middle East respiratory syndrome coronavirus (MERS-CoV) outbreak of 2012 began in the Arabian Peninsula and has been traced through dromedaries to bats [2,7]. MERS-CoV has infected 2,578 persons to date with a case mortality rate of 34.4% [8]. SARS-CoV-1 has not been detected since 2004 [9], whereas MERS-CoV reached a peak in 2015 and has diminished since then [8]. The SARS-CoV-2 pandemic has continued for three years to date; cases plateaued during early 2020 but have increased and oscillated since then [1], notably due to a surge of variant forms of SARS-CoV-2 such as delta, lambda, mu, and omicron [10]. Based on experience with SARS-CoV-1 and MERS-CoV, no accurate prediction of the SARS-CoV-2 pandemic duration is presently possible.

Currently, 25 COVID-19 vaccines have received emergency-use authorizations worldwide [11,12]; these vary in strategy and are RNA-liposomal, adenovirus-vector based, inactivated-virus, or protein-subunit vaccines. Vaccine efficacy is high, ranging from 67 to 95% [13]. Immunization is and will continue to be a very important means to control the SARS-CoV-2 pandemic, but some fraction of the population will not be protected, some individuals may not have access to the vaccine, contraindications may prevent some from being vaccinated, and vaccines prepared to earlier strains of the virus may have diminished efficacy presently. Accordingly, antiviral therapeutics are still required.

A wide variety of approaches have been taken toward the development of therapeutics for SARS-CoV-2 infections. Small-molecule drugs identified include remdesivir, favipiravir, ribavirin, oseltamivir (Tamiflu®), lopinavir, camostat, umifenovir (Arabidiol®), chloroquine, hydroxychloroquine, azithromycin, ivermectin, and glucocorticoids [14-19]. Interferons and immunoglobulins have also been explored [15,17].

Computational approaches have also contributed to our understanding of potentially active compounds that may be used to treat SARS-CoV-2 infections [20]. Specific approaches have included database searches, molecular modeling, and dynamics; targets have included, for example, the SARS-CoV-2 spike glycoprotein (S-protein) and main protease (Mpro, 3CLpro Nsp5) [18,21,22].

Chloroquine and hydroxychloroquine are antimalarials that also have been shown to interfere with the entrance of SARS-CoV-2 into human cells via the acetyl cholinesterase-2 (ACE2) receptor [15,16]. Clinical use of these drugs repurposed against SARS-CoV-2 has been controversial, but 80% of conclusive trials with significant study size were positive, and essentially 100% of early-stage disease studies were favorable. Negative trials were conducted on hospitalized patients with severe SARS-CoV-2 disease [23-26]. Nonetheless, 68% of late-stage studies have also shown efficacy. Thus, it appears that these aminoquinolines are best used with early to mid-stage disease. Adverse effects of hydroxychloroquine have been found recently, and these include lipidosis and podocytopathy [27], but these side effects must be weighed against therapeutic benefit.

Chlorpheniramine maleate (Chlorphenamine, 1-(2-pyridyl)-1-(4-chlorophenyl)-3-dimethylamino propane, SMILES: CN(C)CCC(C1=CC=C(C=C1)Cl)C2=CC=CC=N2) is an over-the-counter (OTC) antihistamine that was first prepared in 1951 [28] and has been in use for over 70 years. It has been found to be safe and effective with minimal side effects such as drowsiness and dry mouth, nose, and throat. Furthermore, it is widely available and is cost-effective. Chlorpheniramine also has been shown to be active as an antiviral against the human Ebola virus [29] and human influenza viruses [30]. The present work explores the activity of chlorpheniramine against SARS-CoV-2 by means of DrugBank structural searches, molecular modeling, and preliminary clinical evidence from a retrospective study.

## Materials and methods

Molecular modeling studies

The present approach to finding new candidate drugs for SARS-CoV-2 infections is somewhat different from others who have used screening or modeling. Rather, drugs of known activity, namely chloroquine and hydroxychloroquine that are broadly active against SARS-CoV-1, MERS-CoV, and SARS-CoV-2 and prevent the entry of the virus into cells, were employed in this study [15,31]. This approach seemed most fruitful as drugs that prevent cellular damage by the virus should be the most effective; few cells would be harmed, and the virus would remain in the bloodstream, respiratory system, or gastrointestinal tract to be detected and neutralized by the immune system or eliminated directly. Furthermore, a drug active against SARS-CoV-2 and readily available OTC was sought for this study.

A three-tiered approach was used in which the chemical structures of chloroquine and hydroxychloroquine were searched in the DrugBank database [32] against 13,580 drugs for related structures with Similarity Threshold = 0.35, molecular weight > 200, and Drug Types = Approved, Veterinary Approved, and Nutraceuticals; structural matches were rescreened against oral OTC drugs [33]; and the ultimately identified drugs were energy minimized with Spartan 10 software [34] (Hartree Fock 6-31-G* basis set in the presence of water with convergence). Energy-minimized structures were compared three-dimensionally to chloroquine, hydroxychloroquine, and chlorpheniramine crystal structures to achieve placement of related functional groups in similar portions of space. The present DrugBank search strategy was unique among others that have been reported and is based on drugs of known efficacy against SARS-CoV-2; the search threshold was set to 0.35 to survey related structures broadly. Energy minimization by this strategy should result in accurate structures equivalent to the conformation of the drugs in an aqueous solution. Software used in modeling also included Avogadro v1.2.0 [35], PubChem 3D Viewer v2.0 [36], LigandScout v4.45 [37], and Mercury v4.0 [38].

Preliminary clinical data collection

A retrospective human clinical study with chlorpheniramine maleate was performed online and participation in this study was entirely voluntary (https://www.surveymonkey.com/r/Q7RCCCV). Participants were recruited by word of mouth during the period of January 10, 2021, to November 16, 2021, and a majority of responses were received between January and February 2021. Fifteen questions were asked to the volunteers in this online questionnaire: 1. Demographic information, 2. Please say how you took chlorpheniramine (choose one option that best describes your situation), 3. What dose of chlorpheniramine did you take? (please select only one), 4. Date of known exposure to the COVID-19 coronavirus? (please leave blank if unknown), 5. Date of your COVID-19 viral antigen test (PCR or other; please leave blank if not tested), 6. Supplements, vitamins, and prescriptions you take, 7. Results of your COVID-19 test? (please leave blank if inapplicable), 8. When did you become ill with COVID-19 and begin to experience symptoms? (please leave blank if inapplicable), 9. Which symptoms did you experience when you were ill with COVID-19? (select all appropriate responses), 10. How ill did you become after you contracted COVID-19? 11. Were you hospitalized? 12. Your comorbidities or conditions (please check all applicable chronic conditions), 13. How many days were you ill with COVID-19? 14. How much do you believe that chlorpheniramine helped during your COVID-19 disease? and 15. Please provide any other information or feedback that you feel would be helpful to this retrospective study.

Lists of responses also included a free-response option, and sliders were provided, when appropriate, to ease response time. Volunteers provided information that covered November 2, 2020, to November 16, 2021, and many provided information anecdotally on persons who had also taken chlorpheniramine and remained healthy but did not complete the survey. Analysis of results was accomplished through online tools provided by the survey company and with Microsoft Excel. The confidentiality of all respondents and their information was protected.

## Results

The results of a *DrugBank* structural search with chloroquine are shown in Table 1. Seventy-two drugs of a similar structure were found, with hydroxychloroquine as the highest score (0.950) and chlorpheniramine as a mid-score (0.377) drug. Fourteen classes of drugs are represented in the matches, including 19 antibiotics, 17 antineoplastics, nine neuroactive drugs, six anesthetics, five antimalarials, three antihistamines, two antifungals, two antiseptics, two anti-inflammatories, two non-steroidal anti-inflammatory drugs (NSAIDs), one anti-asthmatic, one antiemetic, one antirheumatic, and one cardiovascular drug.

**Table 1:** Drug Structures Similar to Chloroquine * * Screened from 13,580 drugs by chemical similarity with chloroquine structure at Threshold = 0.35, molecular weight > 200 g/mol, and Drug Types = Approved, Veterinary Approved, and Nutraceuticals. Drug class abbreviations: AM = antimalarial, AS = antiseptic, AB = antibiotic, AC = antineoplastic, N = neuroactive, AF = antifungal, AV = antiemetic, NS = NSAIDs (non-steroidal anti-inflammatories), CV = cardiovascular drugs, AH = antihistamines, AA = anti-asthmatics, AI = anti-inflammatories, AR = antirheumatics, and AE = anesthetics; NCNPP = N-Cyclohexyl-N'-phenyl-p-phenylenediamine

DrugBank Database Structural Match (class)	Score	DrugBank Database Structural Match (class)	Score
Hydroxychloroquine (AM)	0.950	Chlorpheniramine (AH)	0.377
Amodiaquine (AM)	0.565	Montelukast (AA)	0.376
Primaquine (AM)	0.519	Orbifloxacin (AB)	0.376
Dequalinium (AS)	0.483	Tofacitinib (AR)	0.376
Chlorquinaldol (AS)	0.473	Brimonidine (AI)	0.372
Proflavine (AB)	0.466	Erlotinib (AC)	0.371
Cabozantinib (AC)	0.438	Thenalidine (AH)	0.369
Dacomitinib (AC)	0.429	Sarafloxacin (AB)	0.368
Chloroxine (AB)	0.428	Difloxacin (AB)	0.368
Danofloxacin (AB)	0.419	Pefloxacin (AB)	0.367
Cariprazine (N)	0.419	Norfloxacin (AB)	0.367
Besifloxacin (AB)	0.414	Mepivacaine (AE)	0.365
Gefitinib (AC)	0.411	Degarelix (AC)	0.363
Tafenoquine (AM)	0.409	Ropivacaine (AE)	0.363
Clioquinol (AF)	0.401	Bupivacaine (AE)	0.363
Lenvatinib (AC)	0.399	Levobupivacaine (AE)	0.363
NCNPP	0.397	Pergolide (N)	0.362
Domperidone (AV)	0.396	Mefloquine (AM)	0.362
Antrafenine (NS)	0.394	Boscalid (AF)	0.362
Sertindole (N)	0.391	Clomipramine (N)	0.361
Bosutinib (AC)	0.389	Floctafenine (AI)	0.360
Lomefloxacin (AB)	0.389	Vandetanib (AC)	0.359
Clofazimine (AB)	0.387	Tropisetron (N)	0.359
Sparfloxacin (AB)	0.387	Glasdegib (AC)	0.358
Grepafloxacin (AB)	0.385	Periciazine (AE)	0.356
Neratinib (AC)	0.385	Clobazam (N)	0.355
Amsacrine (AC)	0.383	Bazedoxifene (AC)	0.355
Quinupramine (N)	0.382	Finafloxacin (AB)	0.355
Pradofloxacin (AB)	0.380	Bendamustine (AC)	0.354
Afatinib (AC)	0.379	Etidocaine (AE)	0.354
Ciprofloxacin (AB)	0.379	Trazodone (N)	0.354
Enrofloxacin (AB)	0.379	Carprofen (NS)	0.354
Brexpiprazole (AB)	0.379	Alectinib (AC)	0.353
Imiquimod (AC)	0.378	Delafloxacin (AB)	0.350
Indoramin (CV)	0.378	Dexchlorpheniramine maleate (AH)	0.350
Fentanyl (N)	0.377	Lapatinib (AC)	0.350

A structural search with hydroxychloroquine, as shown in Table 2, found similar results with chloroquine scoring 0.950 and chlorpheniramine scoring 0.371. Drug classes remained the same.

**Table 2:** Drug Structures Similar to Hydroxychloroquine *& * Screened from 13,580 drugs by chemical similarity with hydroxychloroquine structure at Threshold = 0.35, molecular weight > 200 g/mol, and Drug Types = Approved, Veterinary Approved, and Nutraceuticals. Drug class abbreviations: AM = antimalarial, AS = antiseptic, AB = antibiotic, AC = antineoplastic, N = neuroactive, AF = antifungal, AV = antiemetic, NS = NSAIDs (non-steroidal anti-inflammatories), CV = cardiovascular drugs, AH = antihistamines, AA = anti-asthmatics, AI = anti-inflammatories, AR = antirheumatics, and AE = anesthetics; AT = antithrombotic ^&^ 13 differences with respect to the search with chloroquine are indicated as italic entries

DrugBank Database Structural Match (class)	Score	DrugBank Database Structural Match (class)	Score
Chloroquine (AM)	0.950	Pefloxacin (AB)	0.381
Amodiaquine (AM)	0.564	Norfloxacin (AB)	0.381
Primaquine (AM)	0.529	Bazedoxifene (AC)	0.381
Chlorquinaldol (AS)	0.493	Indoramin (CV)	0.381
Dequalinium (AS)	0.474	Erlotinib (AC)	0.380
Proflavine (AB)	0.443	Fentanyl (N)	0.380
Chloroxine (AB)	0.440	Boscalid (AF)	0.379
Gefitinib (AC)	0.439	Remifentanil (AE)	0.378
Dacomitinib (AC)	0.436	Dipyridamole (AT)	0.378
Danofloxacin (AB)	0.433	Tofacitinib (AR)	0.378
Besifloxacin (AB)	0.426	Sufentanil (AE)	0.378
Antrafenine (NS)	0.423	Alectinib (AC)	0.377
Bosutinib (AC)	0.415	Mepivacaine (AE)	0.376
Cariprazine (N)	0.413	Finafloxacin (AB)	0.376
Lenvatinib (AC)	0.407	Ropivacaine (AE)	0.374
Clioquinol (AF)	0.404	Bupivacaine (AE)	0.374
Lomefloxacin (AB)	0.403	Levobupivacaine (AE)	0.374
Sparfloxacin (AB)	0.400	Imiquimod (AC)	0.374
Grepafloxacin (AB)	0.398	Carprofen (NS)	0.373
Afatinib (AC)	0.398	Bendamustine (AC)	0.372
Domperidone (AV)	0.398	Chlorpheniramine (AH)	0.371
Montelukast (AA)	0.396	Perphenazine (N)	0.371
Orbifloxacin (AB)	0.394	Cetrorelix (H)	0.370
Ciprofloxacin (AB)	0.393	Diperodon (AE)	0.370
Enrofloxacin (AB)	0.393	Halofuginone (AS)	0.370
Sertindole (N)	0.393	Vandetanib (AC)	0.368
Pradofloxacin (AB)	0.392	Pindolol (CV)	0.367
Clofazimine (AB)	0.389	Periciazine (AE)	0.366
Floctafenine (AI)	0.389	Trimetrexate (AC)	0.366
Mefloquine (AM)	0.389	Etidocaine (AE)	0.366
Brexpiprazole (AB)	0.387	Thenalidine (AH)	0.364
Amsacrine (AC)	0.386	Vismodegib (AC)	0.364
Sarafloxacin (AB)	0.383	Alfuzosin (AC)	0.363
Difloxacin (AB)	0.383	Carfentanil (N)	0.363
Brimonidine (AI)	0.382	Lapatinib (AC)	0.363
Tropisetron (N)	0.382	Trazodone (N)	0.362

**Figure 1:**
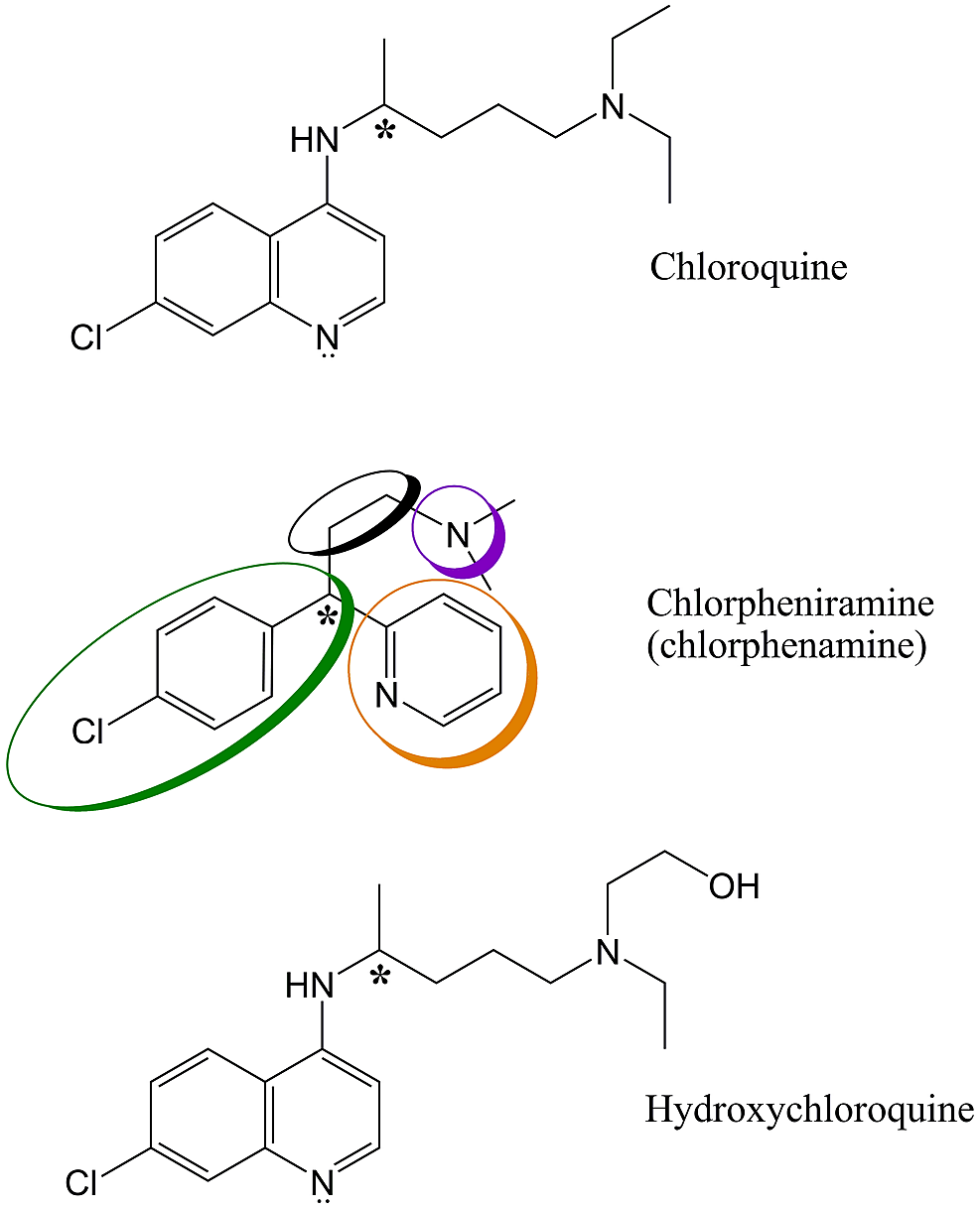
Comparison of Chlorpheniramine structure with those of Chloroquine and Hydroxychloroquine Common structural features are indicated by colored ovals: chlorophenyl group (green), pyridine ring (orange), alkyl sidechain (black), and tertiary amine (purple). * = chiral carbons.

These 72 drugs were screened against oral OTC medications, and only chlorpheniramine and dexchlorpheniramine, both OTC antihistamines, remained. Dexchlorpheniramine is the dextrorotatory isomer or *S*(+)-chlorpheniramine whereas chlorpheniramine maleate is prepared as a racemic mixture of *R* and *S* enantiomers. Thus, only one compound resulted from the OTC screening. The structure of chlorpheniramine is compared with chloroquine and hydroxychloroquine in Figure 1.

Chlorpheniramine shares four common structural features with chloroquine and hydroxychloroquine, namely chlorophenyl group, pyridine ring, tertiary amine, and alkyl sidechain. Structural differences include the presence of a secondary amine in chloroquine and hydroxychloroquine that chlorpheniramine lacks, fused chlorophenyl and pyridine rings (quinoline ring) in chloroquine and hydroxychloroquine, and longer alkyl sidechain and tertiary amine substituents in chloroquine and hydroxychloroquine.

Some properties of the screened drugs are compared in Table 3. Log(*P*)_OW_ (octanol:water partition coefficient) values for the drugs shown in Table 3 are strongly positive which indicates significant hydrophobicity; in this respect, the log(*P*) of hydroxychloroquine is closer to that of chlorpheniramine than it is to that of chloroquine. Chlorpheniramine and dexchlorpheniramine are considerably more water soluble than chloroquine and hydroxychloroquine. Lastly, the p*K*a of hydroxychloroquine lies midway between those of chloroquine and chlorpheniramine.

**Table 3:** Properties of Final Drugs Under Study * * Data were obtained from PubChem.com, DrugBank.com, and the present work. The first molecular weight column is for the free base form of the drugs; the second is for the molecular weight of the maleate salt for the chlorpheniramine compounds. Log(*P*)_ow_ refers to the octanol:water partition coefficient.

Drug	CAS Number	DrugBank code	MW (g/mol)	MW (maleate)	log(P)_OW_	H_2_O Solubility (mg/L)	pK_a_
Chloroquine	54-05-7	DB00608	319.18	-	4.63	0.14	10.1
Hydroxychloroquine	118-42-3	DB01611	335.18	-	3.87	0.026	9.67
Chlorpheniramine	132-22-9	DB01114	274.79	390.14	3.38	160	9.13
Dexchlopheniramine	25523-97-1	DB09555	274.79	390.14	3.39	>100	9.33

*S*-Chlorpheniramine, *R*-hydroxychloroquine, and *R*-chloroquine were chosen for further study as these enantiomers are known to be pharmacologically active [39,40]. The structures were energy minimized in the presence of water, and the final structures were aligned by the chlorophenyl ring, a common structural feature and a known hydrophobic pharmacophore of chloroquine, hydroxychloroquine, and chlorpheniramine [41]. Pharmacophores are molecular portions of the drug that confer biological activity when bound to a target macromolecule.

**Figure 2:**
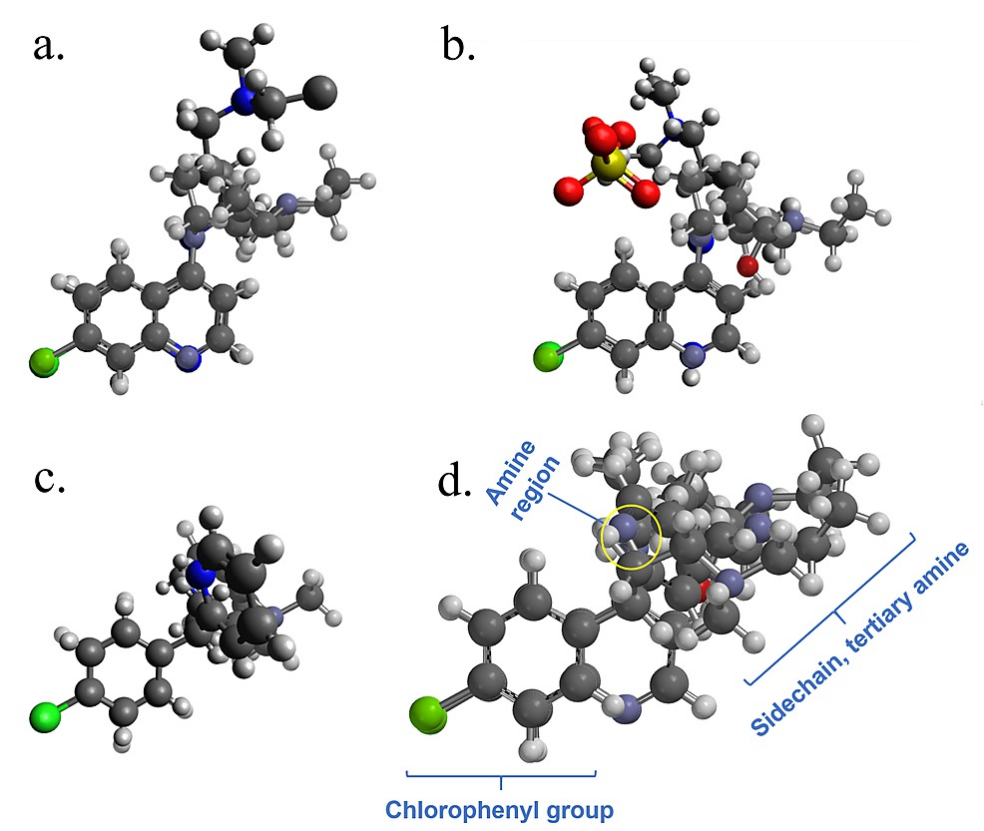
Aligned three-dimensional structures of Chloroquine, Hydroxychloroquine, and Chlorpheniramine Energy-minimized structures of *R*-chloroquine, *R*-hydroxychloroquine, and *S*-chlorpheniramine are shown with silver bonds; crystal structures of *S*-chloroquine (CDMQUI), *R*-hydroxychloroquine sulfate (QOBHUL), and *R*-chlorpheniramine maleate (JEGWUN) are shown with black bonds. Color coding for atoms: carbon, black; nitrogen, blue; hydrogen, white; chlorine, green; oxygen, red; sulfur, yellow. a. *R*-Chloroquine: energy minimized (front) aligned with the crystal structure. b. *R*-Hydroxychloroquine: energy minimized (front) aligned with the crystal structure. c. *S*-Chlorpheniramine: energy-minimized structure aligned with *R*-chlorpheniramine crystal structure (front). d. *R*-Chloroquine, *R*-hydroxychloroquine, and *S*-chlorpheniramine (front) aqueous energy-minimized structures aligned.

Comparison of the *R*-chloroquine energy-minimized structure to the crystal structure [42] in Figure 2a shows the alignment of the quinoline rings and secondary amines, but differing conformations for the alkyl sidechains with tertiary amines; the alkyl chain is slightly forward and right in energy-minimized chloroquine whereas it projects up, forward, and centered in the crystal structure. The same is true of hydroxychloroquine (Figure 2b), but the sidechain in the crystal structure [42] projects up, forward, and left compared to the energy-minimized structure which assumes a conformation like that of energy-minimized chloroquine (Figure 2a). Comparison of energy-minimized *S*-chlorpheniramine to the *R*-chlorpheniramine crystal structure [42,43] (Figure 2c) shows the overlap of the chlorophenyl groups and benzyl carbons, but the configuration of the alkyl chains and pyridine rings are, as expected, opposite one another; *S*-chlorpheniramine has the alkyl sidechain to the right compared to the *R*-isomer in which the sidechain projects backward. The pyridine ring of *S*-chlorpheniramine is behind the chlorophenyl ring with the nitrogen atom pointing up, whereas the pyridine ring in the *R*-isomer projects forward.

The energy-minimized structures are all similar, but the crystal structures vary in significant ways from each other as well as from the energy-minimized structures. The crystal structures were obtained from organic solvents (*e.g.* ethanol, ethyl acetate, DMSO) [44-46], whereas energy-minimization was performed in the presence of water. Thus, the energy-minimized structures appear to be more reliable representations of the aqueous behavior of chloroquine, hydroxychloroquine, and chlorpheniramine. The energy-minimized structures of these three drugs are aligned in Figure 2d. The chlorophenyl groups and equivalent “benzyl” carbons show a near-exact correspondence in all structures. Interestingly, the pyridine nitrogen of chlorpheniramine is very close in space to the secondary amines of chloroquine and hydroxychloroquine (“amine region”), and the alkyl sidechains and tertiary amines are clustered with nitrogen atoms in similar regions of space (“sidechain, tertiary amine”). This suggests that not only do these molecules have many structural elements in common, but they also share similar three-dimensional structural features.

For the present work, a retrospective clinical study was performed on 13 human participants who took chlorpheniramine (one to three 4 mg tablets per day) either preventively (78.6%) or post-exposure (21.4%). Out of the total participants, 54% had comorbidities (*e.g.* asthma, hypertension, Lyme disease, and blood-clotting disorders), 63% tested positive for the virus, and 38% became ill with COVID-19 disease (fatigue, sore throat, fever, chills, cough, shortness of breath, difficulty breathing, muscle ache, loss of taste and smell, and congestion). Preliminary results showed that no participant was hospitalized, and none died. Participants with COVID-19 disease recovered in an average of 7.8 ± 5.0 days, and respondents believed that chlorpheniramine had helped them an average of 65%.

## Discussion

The strategy of searching for chemical structures related to those of chloroquine and hydroxychloroquine followed by screening results against oral, over-the-counter drugs yielded only chlorpheniramine. In other words, chlorpheniramine represents the only OTC drug that can be considered a possible therapeutic agent against SARS-CoV-2 to prevent its entry into human cells. This antihistamine has already been shown to possess antiviral action against the Ebola virus [29] and Influenza viruses [30] which supports its suggested use against the SARS-CoV-2 virus.

Energy minimization in the presence of water in conjunction with molecular modeling and alignment showed that all three drugs are similar three-dimensionally and, thus, may act equivalently against SARS-CoV-2 and other viruses. The three identified regions (chlorophenyl group, “amine region”, and alkyl “sidechain, tertiary amine” region) may be important as possible pharmacophores. In studies with the Ebola virus, four hydrophobic interactions, which encompass the above three regions, were important pharmacophores of chloroquine [43].

Hydroxychloroquine appears to exhibit greater efficacy against SARS-CoV-2 than does chloroquine [15]. The results presented here show that chlorpheniramine shares properties with both aminoquinolines, but it more closely resembles hydroxychloroquine with which it shares similar log(*P*) and p*K*_a_ values. This means that Chlorpheniramine and hydroxychloroquine are closely related by hydrophobicity and acid-base properties, both of which are known to be of significant importance in drug-receptor interactions. In addition, the three-dimensional structure of chlorpheniramine is more related to hydroxychloroquine than it is to chloroquine. Thus, with greater resemblance to the more active drug hydroxychloroquine, chlorpheniramine is more likely to have efficacy against SARS-CoV-2. *In silico* molecular-dynamics calculations would be a useful complement to these results.

A recent clinical study from the University of Utah examined chlorpheniramine maleate nasal spray as a possible treatment for SARS-CoV-2 [47]; they found a 99.7% reduction of viral load after 25 min of treatment. This provides additional support for the conclusions of the present work. It is also in harmony with the preliminary retrospective clinical findings presented in this article. Clearly, prospective, double-blinded, placebo-controlled, randomized clinical studies with chlorpheniramine and dexchlorpheniramine will be important to establish firm pharmacologic links between the drug, the active enantiomer, and treatment of COVID-19 disease.

## Conclusions

Present results from structural database searches, aqueous energy-minimized structure three-dimensional analyses, and preliminary clinical findings indicate that chlorpheniramine maleate, an inexpensive and widely available antihistamine, possesses antiviral activity against SARS-CoV-2.

## Appendices

Appendix 1. Three-dimensional structure of *R*-chloroquine (R-Chloroquine aqueous.pdb) by energy minimization in the presence of water. This structure can be viewed by copying the text below and pasting it into a plain text file with the extension set as .pdb; the resulting file can be read with most 3D software.

HEADER REMARK Spartan '10 exported M0001 R-Chloroquine aqueous.pdb HETATM    1  C   UNK  0001      -4.865  -0.979  -0.247 HETATM    2  H   UNK  0001      -5.034   0.024  -2.112 HETATM    3  C   UNK  0001      -4.491  -0.087  -1.195 HETATM    4  C   UNK  0001      -3.053  -0.349   1.175 HETATM    5  C   UNK  0001      -3.351   0.730  -0.981 HETATM    6  C   UNK  0001      -4.144  -1.127   0.958 HETATM    7  C   UNK  0001      -2.626   0.610   0.218 HETATM    8  H   UNK  0001      -4.462  -1.851   1.682 HETATM    9  H   UNK  0001      -2.504  -0.476   2.088 HETATM   10  C   UNK  0001      -1.975   2.349  -1.768 HETATM   11  H   UNK  0001      -1.723   3.035  -2.557 HETATM   12  C   UNK  0001      -1.187   2.331  -0.600 HETATM   13  H   UNK  0001      -0.369   3.016  -0.498 HETATM   14  C   UNK  0001      -1.490   1.463   0.405 HETATM   15  N   UNK  0001      -3.013   1.598  -1.969 HETATM   16 Cl   UNK  0001      -6.268  -1.986  -0.504 HETATM   17  N   UNK  0001      -0.717   1.439   1.587 HETATM   18  H   UNK  0001      -1.311   1.280   2.377 HETATM   19  C   UNK  0001       0.421   0.495   1.643 HETATM   20  H   UNK  0001       0.121  -0.471   1.235 HETATM   21  H   UNK  0001      -0.069  -0.058   3.681 HETATM   22  C   UNK  0001       0.783   0.303   3.112 HETATM   23  H   UNK  0001       1.575  -0.426   3.229 HETATM   24  H   UNK  0001       1.110   1.239   3.553 HETATM   25  C   UNK  0001       1.599   1.015   0.816 HETATM   26  H   UNK  0001       1.253   1.226  -0.190 HETATM   27  H   UNK  0001       1.927   1.962   1.237 HETATM   28  C   UNK  0001       2.780   0.043   0.722 HETATM   29  H   UNK  0001       2.420  -0.927   0.389 HETATM   30  H   UNK  0001       3.234  -0.109   1.695 HETATM   31  H   UNK  0001       4.200   1.520   0.150 HETATM   32  C   UNK  0001       3.856   0.564  -0.228 HETATM   33  H   UNK  0001       3.415   0.762  -1.209 HETATM   34  N   UNK  0001       5.017  -0.313  -0.338 HETATM   35  H   UNK  0001       3.676  -1.552  -1.375 HETATM   36  C   UNK  0001       4.710  -1.562  -1.048 HETATM   37  H   UNK  0001       5.296  -1.624  -1.960 HETATM   38  C   UNK  0001       6.157   0.391  -0.916 HETATM   39  H   UNK  0001       5.984   0.627  -1.970 HETATM   40  H   UNK  0001       6.254   1.339  -0.401 HETATM   41  C   UNK  0001       7.478  -0.355  -0.777 HETATM   42  H   UNK  0001       7.480  -1.300  -1.308 HETATM   43  H   UNK  0001       7.703  -0.552   0.266 HETATM   44  H   UNK  0001       8.281   0.248  -1.188 HETATM   45  H   UNK  0001       4.334  -2.812   0.679 HETATM   46  C   UNK  0001       4.950  -2.814  -0.213 HETATM   47  H   UNK  0001       4.711  -3.705  -0.788 HETATM   48  H   UNK  0001       5.985  -2.885   0.103 CONECT    1    3    6   16 CONECT    2    3 CONECT    3    2    1    5 CONECT    4    6    7    9 CONECT    5    3    7   15 CONECT    6    1    4    8 CONECT    7    5    4   14 CONECT    8    6 CONECT    9    4 CONECT   10   12   11   15 CONECT   11   10 CONECT   12   10   14   13 CONECT   13   12 CONECT   14    7   12   17 CONECT   15   10    5 CONECT   16    1 CONECT   17   18   14   19 CONECT   18   17 CONECT   19   20   17   22   25 CONECT   20   19 CONECT   21   22 CONECT   22   21   23   24   19 CONECT   23   22 CONECT   24   22 CONECT   25   26   27   19   28 CONECT   26   25 CONECT   27   25 CONECT   28   29   30   25   32 CONECT   29   28 CONECT   30   28 CONECT   31   32 CONECT   32   31   33   28   34 CONECT   33   32 CONECT   34   32   36   38 CONECT   35   36 CONECT   36   35   37   34   46 CONECT   37   36 CONECT   38   39   40   34   41 CONECT   39   38 CONECT   40   38 CONECT   41   42   43   44   38 CONECT   42   41 CONECT   43   41 CONECT   44   41 CONECT   45   46 CONECT   46   45   47   48   36 CONECT   47   46 CONECT   48   46 END
 

Appendix 2. Three-dimensional structure of *R*-hydroxychloroquine (R-Hydroxychloroquine aqueous.pdb) by energy minimization in the presence of water. This structure can be viewed by copying the text below and pasting it into a plain text file with the extension set as .pdb; the resulting file can be read with most 3D software.

HEADER REMARK Spartan '10 exported M0001 R-Hydroxychloroquine aqueous.pdb HETATM    1  C   UNK  0001      -5.123  -0.826  -0.333 HETATM    2  H   UNK  0001      -5.264   0.356  -2.092 HETATM    3  C   UNK  0001      -4.725   0.144  -1.190 HETATM    4  C   UNK  0001      -3.297  -0.383   1.141 HETATM    5  C   UNK  0001      -3.565   0.907  -0.900 HETATM    6  C   UNK  0001      -4.407  -1.109   0.851 HETATM    7  C   UNK  0001      -2.844   0.653   0.281 HETATM    8  H   UNK  0001      -4.744  -1.892   1.502 HETATM    9  H   UNK  0001      -2.752  -0.613   2.036 HETATM   10  C   UNK  0001      -2.145   2.556  -1.531 HETATM   11  H   UNK  0001      -1.874   3.307  -2.251 HETATM   12  C   UNK  0001      -1.360   2.406  -0.371 HETATM   13  H   UNK  0001      -0.525   3.057  -0.205 HETATM   14  C   UNK  0001      -1.687   1.455   0.548 HETATM   15  N   UNK  0001      -3.203   1.855  -1.800 HETATM   16 Cl   UNK  0001      -6.550  -1.768  -0.685 HETATM   17  N   UNK  0001      -0.921   1.302   1.724 HETATM   18  H   UNK  0001      -1.521   1.069   2.491 HETATM   19  C   UNK  0001       0.208   0.346   1.691 HETATM   20  H   UNK  0001      -0.099  -0.574   1.192 HETATM   21  H   UNK  0001      -0.293  -0.392   3.667 HETATM   22  C   UNK  0001       0.565   0.011   3.136 HETATM   23  H   UNK  0001       1.346  -0.735   3.182 HETATM   24  H   UNK  0001       0.903   0.897   3.664 HETATM   25  C   UNK  0001       1.394   0.929   0.918 HETATM   26  H   UNK  0001       1.058   1.210  -0.074 HETATM   27  H   UNK  0001       1.714   1.843   1.412 HETATM   28  C   UNK  0001       2.579  -0.033   0.770 HETATM   29  H   UNK  0001       2.218  -0.989   0.407 HETATM   30  H   UNK  0001       3.051  -0.204   1.730 HETATM   31  H   UNK  0001       3.817   1.557   0.115 HETATM   32  C   UNK  0001       3.610   0.540  -0.199 HETATM   33  H   UNK  0001       3.157   0.611  -1.187 HETATM   34  N   UNK  0001       4.909  -0.136  -0.255 HETATM   35  C   UNK  0001       4.887  -1.539  -0.651 HETATM   36  H   UNK  0001       5.859  -1.748  -1.078 HETATM   37  C   UNK  0001       5.842   0.641  -1.081 HETATM   38  H   UNK  0001       5.698   0.441  -2.145 HETATM   39  H   UNK  0001       5.612   1.689  -0.945 HETATM   40  C   UNK  0001       7.309   0.438  -0.718 HETATM   41  H   UNK  0001       7.640  -0.585  -0.854 HETATM   42  H   UNK  0001       7.487   0.711   0.316 HETATM   43  H   UNK  0001       7.931   1.066  -1.347 HETATM   44  H   UNK  0001       3.729  -2.442   0.957 HETATM   45  C   UNK  0001       4.703  -2.517   0.497 HETATM   46  H   UNK  0001       4.816  -3.529   0.122 HETATM   47  H   UNK  0001       5.456  -2.344   1.255 HETATM   48  O   UNK  0001       3.903  -1.847  -1.619 HETATM   49  H   UNK  0001       4.118  -1.418  -2.440 CONECT    1    3    6   16 CONECT    2    3 CONECT    3    2    1    5 CONECT    4    6    7    9 CONECT    5    3    7   15 CONECT    6    1    4    8 CONECT    7    5    4   14 CONECT    8    6 CONECT    9    4 CONECT   10   12   11   15 CONECT   11   10 CONECT   12   10   14   13 CONECT   13   12 CONECT   14    7   12   17 CONECT   15   10    5 CONECT   16    1 CONECT   17   18   14   19 CONECT   18   17 CONECT   19   20   17   22   25 CONECT   20   19 CONECT   21   22 CONECT   22   21   23   24   19 CONECT   23   22 CONECT   24   22 CONECT   25   26   27   19   28 CONECT   26   25 CONECT   27   25 CONECT   28   29   30   25   32 CONECT   29   28 CONECT   30   28 CONECT   31   32 CONECT   32   31   33   28   34 CONECT   33   32 CONECT   34   32   35   37 CONECT   35   36   34   45   48 CONECT   36   35 CONECT   37   38   39   34   40 CONECT   38   37 CONECT   39   37 CONECT   40   41   42   43   37 CONECT   41   40 CONECT   42   40 CONECT   43   40 CONECT   44   45 CONECT   45   44   46   47   35 CONECT   46   45 CONECT   47   45 CONECT   48   35   49 CONECT   49   48 END

Appendix 3. Three-dimensional structure of *S*-chlorpheniramine (S-Chlorpheniramine aqueous.pdb) by energy minimization in the presence of water. This structure can be viewed by copying the text below and pasting it into a plain text file with the extension set as .pdb; the resulting file can be read with most 3D software.

HEADER REMARK Spartan '10 exported M0001 S-Chlorpheniramine aqueous.pdb HETATM    1  H   UNK  0001       1.169  -0.710   1.854 HETATM    2  C   UNK  0001       1.694  -0.262   1.032 HETATM    3  C   UNK  0001       3.083   0.857  -1.085 HETATM    4  C   UNK  0001       0.979   0.266  -0.041 HETATM    5  C   UNK  0001       3.076  -0.235   1.055 HETATM    6  C   UNK  0001       3.761   0.327  -0.008 HETATM    7  C   UNK  0001       1.697   0.822  -1.091 HETATM    8  H   UNK  0001       3.616  -0.645   1.887 HETATM    9  H   UNK  0001       1.175   1.239  -1.933 HETATM   10  H   UNK  0001       3.623   1.291  -1.906 HETATM   11 Cl   UNK  0001       5.511   0.365   0.018 HETATM   12  C   UNK  0001      -0.548   0.266  -0.079 HETATM   13  H   UNK  0001      -0.833   0.774  -0.990 HETATM   14  C   UNK  0001      -1.139   1.065   1.098 HETATM   15  H   UNK  0001      -0.925   0.551   2.027 HETATM   16  H   UNK  0001      -0.624   2.018   1.152 HETATM   17  H   UNK  0001      -3.142   0.317   1.091 HETATM   18  C   UNK  0001      -2.651   1.281   1.037 HETATM   19  H   UNK  0001      -2.947   1.827   1.938 HETATM   20  N   UNK  0001      -3.141   1.958  -0.158 HETATM   21  H   UNK  0001      -2.956   3.928   0.629 HETATM   22  C   UNK  0001      -2.670   3.330  -0.240 HETATM   23  H   UNK  0001      -1.593   3.365  -0.332 HETATM   24  H   UNK  0001      -3.083   3.804  -1.122 HETATM   25  H   UNK  0001      -4.947   0.908  -0.176 HETATM   26  C   UNK  0001      -4.593   1.932  -0.181 HETATM   27  H   UNK  0001      -5.041   2.448   0.671 HETATM   28  H   UNK  0001      -4.957   2.402  -1.087 HETATM   29  H   UNK  0001      -1.571  -1.006  -2.257 HETATM   30  C   UNK  0001      -1.576  -1.635  -1.388 HETATM   31  C   UNK  0001      -1.566  -3.132   0.852 HETATM   32  C   UNK  0001      -2.052  -2.931  -1.455 HETATM   33  C   UNK  0001      -1.104  -1.149  -0.175 HETATM   34  C   UNK  0001      -2.049  -3.705  -0.307 HETATM   35  H   UNK  0001      -2.421  -3.328  -2.383 HETATM   36  H   UNK  0001      -2.409  -4.716  -0.306 HETATM   37  N   UNK  0001      -1.107  -1.892   0.924 HETATM   38  H   UNK  0001      -1.547  -3.693   1.769 CONECT    1    2 CONECT    2    1    5    4 CONECT    3    6    7   10 CONECT    4    7    2   12 CONECT    5    2    6    8 CONECT    6    5    3   11 CONECT    7    3    4    9 CONECT    8    5 CONECT    9    7 CONECT   10    3 CONECT   11    6 CONECT   12   13    4   14   33 CONECT   13   12 CONECT   14   15   16   12   18 CONECT   15   14 CONECT   16   14 CONECT   17   18 CONECT   18   17   19   14   20 CONECT   19   18 CONECT   20   18   22   26 CONECT   21   22 CONECT   22   21   23   24   20 CONECT   23   22 CONECT   24   22 CONECT   25   26 CONECT   26   25   27   28   20 CONECT   27   26 CONECT   28   26 CONECT   29   30 CONECT   30   29   33   32 CONECT   31   34   37   38 CONECT   32   34   30   35 CONECT   33   30   37   12 CONECT   34   31   32   36 CONECT   35   32 CONECT   36   34 CONECT   37   31   33 CONECT   38   31 END
